# Passive shoulder occupational exoskeleton reduces shoulder muscle coactivation in repetitive arm movements

**DOI:** 10.1038/s41598-024-78090-2

**Published:** 2024-11-13

**Authors:** Lorenzo Grazi, Emilio Trigili, Michele Fiore, Francesco Giovacchini, Angelo Maria Sabatini, Nicola Vitiello, Simona Crea

**Affiliations:** 1 The BioRobotics Institute, Scuola Superiore Sant’Anna, 56025 Pontedera, Pisa, Italy; 2 Department of Excellence in Robotics & AI, Scuola Superiore Sant’Anna, 56127 Pisa, Italy; 3 IUVO S.R.L., 56025 Pontedera, Pisa, Italy; 4 IRCCS Fondazione Don Carlo Gnocchi, 50143 Florence, Italy

**Keywords:** Biomedical engineering, Engineering

## Abstract

Humans naturally employ muscle coactivation to facilitate a broad range of movements, enhancing joint stability and movement accuracy. However, excessive muscle coactivation can become unfavorable or even detrimental. This phenomenon is often observed in industrial workers who endure repetitive or prolonged joint stress, particularly in areas such as the shoulders. Prolonged stress can result in soft tissue damage and the onset of work-related musculoskeletal disorders (MSDs). In recent years, there have been efforts to mitigate the emergence of work-related MSDs among industrial workers through the implementation of upper-limb occupational exoskeletons (OEs). While previous research has demonstrated their effectiveness in reducing shoulder muscle activation, particularly in static and overhead work activities, there has been a lack of studies examining the impact of upper-limb OEs on muscle coactivation during repetitive arm movements. To bridge this gap in knowledge, our study systematically assesses the influence of a passive exoskeleton’s anti-gravitational support on shoulder muscle coactivation during repetitive arm movements. Results show that peak and mean coactivation levels linearly decrease with the increase of the amount of anti-gravitational support provided by the upper-limb OE, reaching approximately 51% and 54%, respectively. Conversely, the percentage of the movement cycle corresponding to the coactivation peak appears unaffected by the level of assistance. This study marks the first instance in which a passive upper-limb OE has been shown to reduce shoulder muscle coactivations, potentially paving the way for a novel methodology in their evaluation.

## Introduction

Muscle coactivation is one of the mechanisms used by the central nervous system (CNS) to improve joint stabilization and movement accuracy, by the simultaneous activation of pairs of agonist–antagonist muscles^1–4^. Muscles can be seen as unidirectional actuators capable of generating only pulling forces, therefore to generate movements every human joint is provided with at least a pair of agonist and antagonist muscles, where the agonists produce forces (hence moments around joints) in the direction of a certain movement, while the antagonists produce forces in the opposite direction^5,6^. To improve joint stability and movement accuracy, muscle coactivation increases the joint apparent stiffness, which is, in linear approximation, given by the summation of the stiffnesses of all muscles acting in parallel on that joint^5^. However, when antagonist muscles generate excessive negative work around a joint, muscle coactivation may become functionally unfavorable, leading to increased human movement inefficiency^6^ and higher metabolic cost^4^. Additionally, excessive muscle coactivation may also increase shear and compression forces on the human joints, which in turn can lead to cartilage loss and eventually cause the development of musculoskeletal disorders (MSDs), especially at workplaces where workers are usually exposed to repetitive or prolonged loading on the joints^4,7^. Such MSDs associated with work activities are called work-related MSDs^8^.

Work-related MSDs are one of the main causes of occupational health problems in the European Union, where three out of five workers are affected^9^. In many countries, work-related MSDs cause the largest percentage of work days lost and contribute to the development of more physical disability than any other group of illnesses^10,11^. Among all the possible work-related MSDs, the most disabling includes shoulder disorders, which involve about 23% of European industrial workers^12^. Such disorders can affect tendons, ligaments, and muscles crossing the shoulder joint, causing, among others, rotator cuff tendinopathies and shoulder impingement syndromes^13^, which are usually correlated with the prolonged maintenance of the arms in poorly ergonomic postures, such as overhead, or with the exposure to repetitive arm gestures, such as manipulating objects and tools of different weights^9,14^. In the last years, to alleviate workers physical burden and possibly reduce the insurgence of work-related MSDs, occupational exoskeletons (OEs) have been proposed as a potentially effective technological tool^15^ to be used when full automation of work tasks is not feasible^16^ or in case human presence is still necessary^17–19^. Notably, while the short-term effectiveness of OEs have been extensively studied^16,20,21^, middle- and long-term effects on human biomechanics still lacks clear evidences^22,23^, despite a few preliminary studies have started investigating these aspects^24^. The absence of conclusive long-term studies is why standards for ergonomic risk assessment tools, such as ISO 11228–3^25^ have not yet been updated to reflect changes in risk when using an exoskeleton; however, some assessment tools have already been revised to account for this potential impact ^26–28^.

OEs are usually defined as personal assistive devices aimed at reducing the physical load on workers who perform demanding activities, through a synergistic action with their users^29^. Among them, most are designed to support the upper extremities, in particular the shoulder joint^20^. Shoulder OEs are more frequently passive devices, although also active^30–32^ and semi-active^33,34^ exoskeletons have been developed. Passive OEs typically rely on elastic elements, like springs, to store and release mechanical energy in specific phases of the movement, they are often designed to be lightweight and do not require a power source to operate, which can be more comfortable for the user to wear for prolonged periods. They also have a lower mechatronic complexity than active and semi-active devices, which use motors to assist the user’s motion and also need electronics and batteries to operate.

**Fig. 1:**
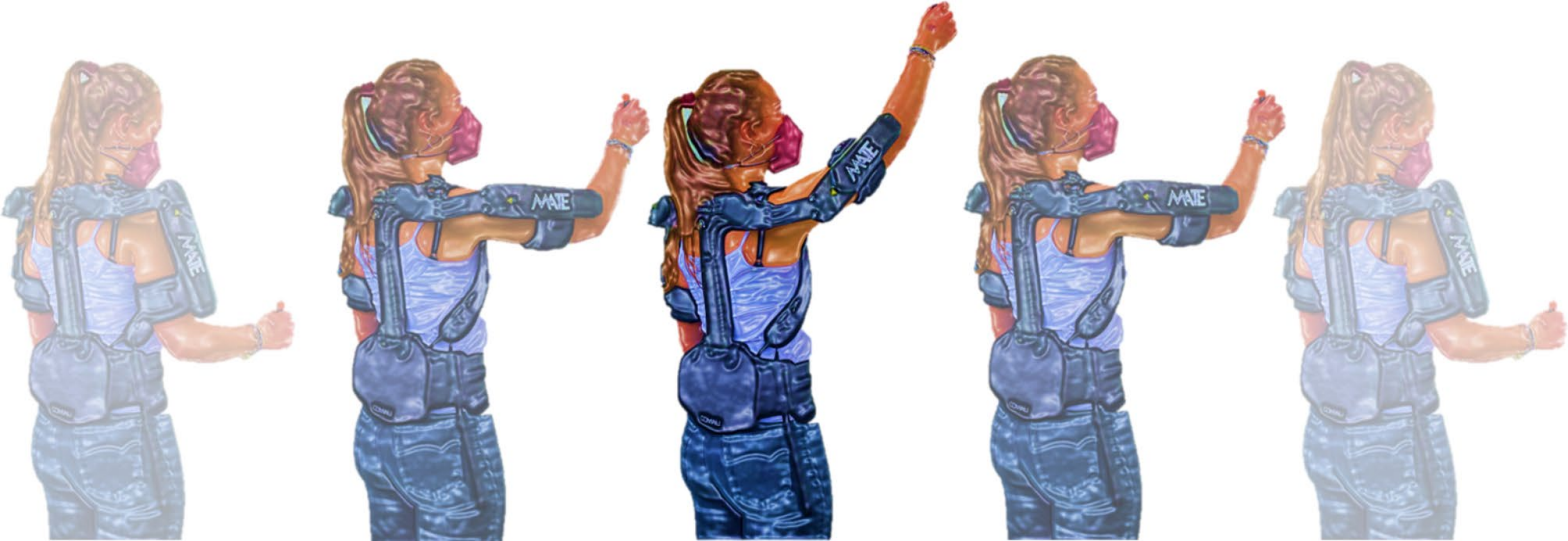
One representative subject wearing the passive upper-limb occupational exoskeleton while executing a sequence of repetitive arm movements. The study aimed to evaluate shoulder muscles coactivation when using a spring-loaded exoskeleton providing anti-gravitational support.

**Fig. 2:**
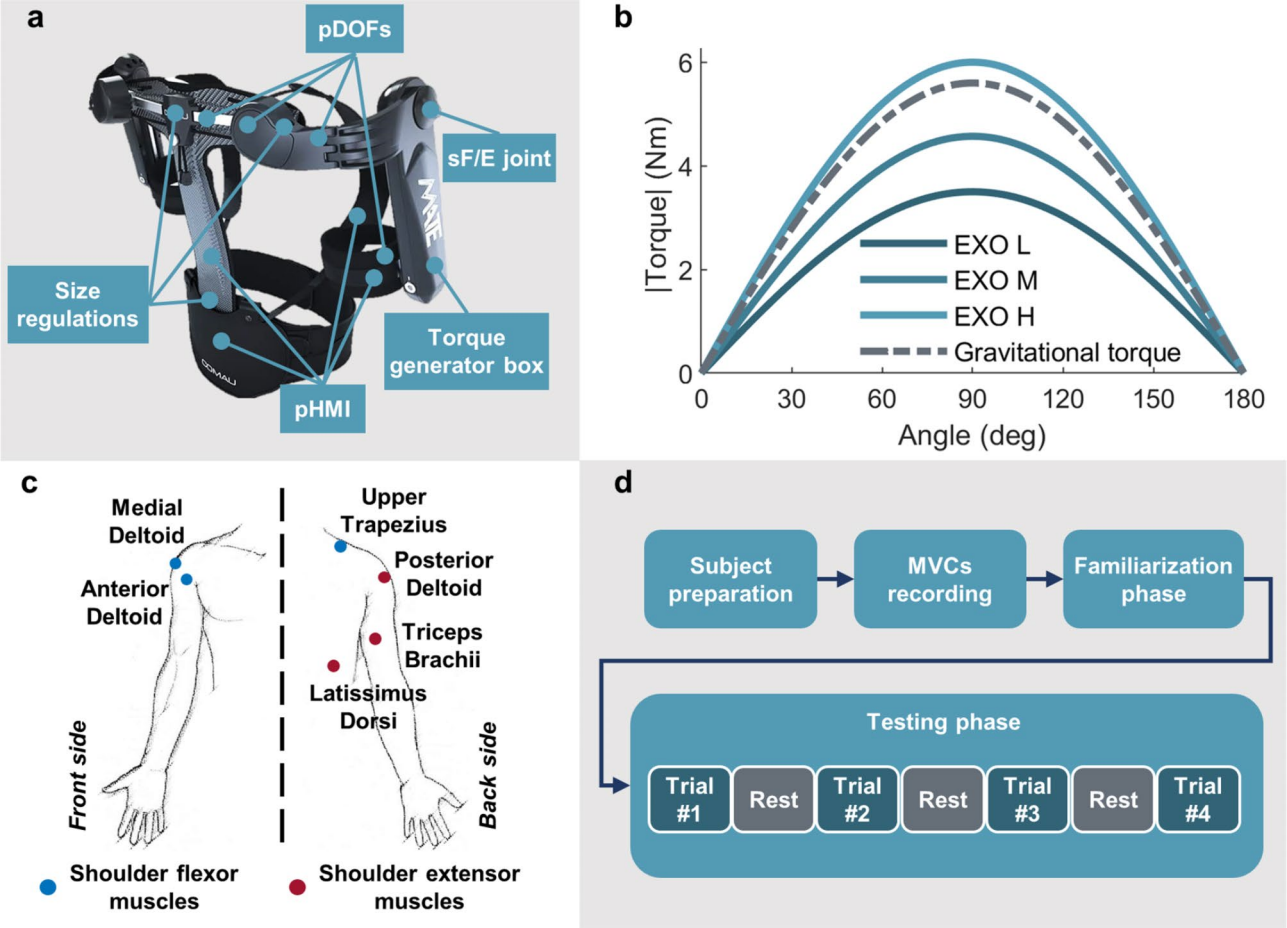
The upper-limb exoskeleton and experimental setup. **(a)** The main components of the exoskeleton are the torque generator boxes, the physical human–machine interface (pHMI), and the kinematic chain of passive degrees of freedom (pDOFs). A number of size regulations allow users with different anthropometries to wear the device. **(b)** The different assistive torque profiles (EXO L, EXO M, EXO H) and the average gravitational torque profile estimated from the subjects of this study are shown. Torque values are reported as absolute values (EXO and NO EXO profiles have opposite sign). **(c)** Schematic representation of EMG sensors location over the arm flexor muscles (Anterior Deltoid, Medial Deltoid, Upper Trapezius) and arm extensor muscles (Posterior Deltoid, Latissimus Dorsi, Triceps Brachii). **(d)** Experimental protocol including subject preparation, MVCs recording, familiarization, and testing phases.

In this work, we aimed to investigate the effect of a passive upper-limb OE on shoulder muscle coactivation while executing repetitive dynamic arm flexion–extension (F/E) movements. Specifically, the subjects were instructed to repetitively perform a pointing task while holding a lightweight screwdriver, moving it from waist level to overhead and vice versa (Fig. 1). We conducted an experimental protocol in which eleven female subjects were requested to perform shoulder flexion and extension movements under different experimental conditions, namely once without wearing the exoskeleton (NO EXO condition) and three times while wearing the exoskeleton providing different levels of anti-gravitational support, namely, low, medium, and high (EXO L, EXO M, EXO H), as shown in Fig. 2. The range of movement was approximately 100 degrees to simulate overhead tasks, and a fixed pace of 50 bpm was maintained using auditory beats from a metronome to minimize movement variability both within and between subjects. Each beat indicated to subjects the start of a new shoulder F/E movement cycle. Electromyographic (EMG) activity from six superficial shoulder muscles was collected and processed to extract muscle coactivation indices based on the time-varying multi-muscle coactivation function ($$TMCf$$)^4,35^. The $$TMCf$$ allowed to take into account the contribution of multiple muscles without the need to a-priori classify the muscles as agonists or antagonists according to the generated moment around the shoulder joint, as in other methods^36–39^. In this study, three balanced configurations, i.e., $$TMCf$$ curves computed considering from one up to three couples of flexor/extensor shoulder muscles, were evaluated. The shoulder flexor muscles were the Anterior Deltoid (AD), Medial Deltoid (MD), and Upper Trapezius (UT), whereas the extensor muscles were the Posterior Deltoid (PD), Triceps Brachii (TB), and Latissimus Dorsi (LD). For each shoulder flexion–extension (sF/E) movement, three coactivation indices were extracted from each $$TMCf$$ curve: (1) the peak value ($${TMCf}_{max}$$), representing the maximum coactivation level over a sF/E cycle; (2) the mean value ($${TMCf}_{mean}$$), representing the average coactivation level over a sF/E cycle; (3) the percentage of the sF/E cycle corresponding to the peak value ($${TMCf}_{phase})$$.

The novelty of this work is twofold. First, it presents the first systematic analysis of muscle coactivations in the context of OE use, demonstrating its potential to enhance understanding of the biomechanical effects of the technology. Second, while most existing studies have primarily explored the effects of upper-limb OEs during static or quasi-static tasks, this research uniquely targets dynamic, repetitive upper-limb movements^40^.

## Results

Variations in muscle coactivation were investigated by examining the impact of (1) the muscle configurations considered and of (2) the assistance levels provided by the exoskeleton on the different coactivation indices. Repeated measures one-way analysis of variance (ANOVA) was carried out on the two factors, separately.

**Fig. 3:**
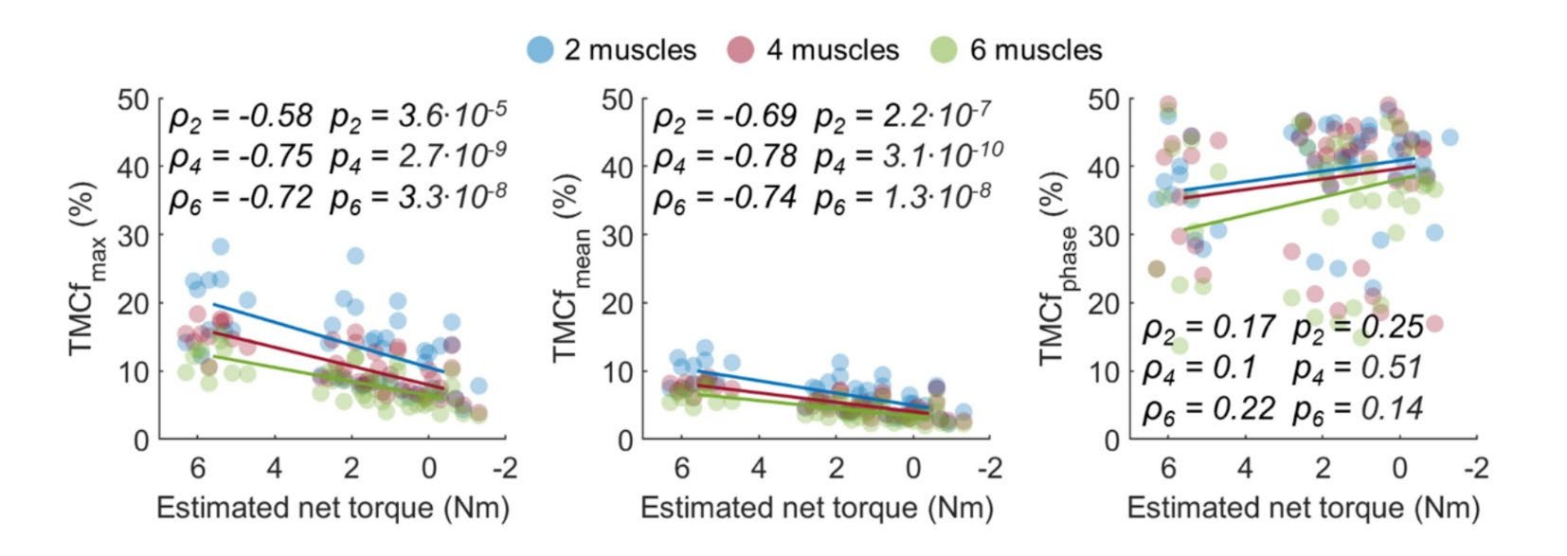
Comparison of the coactivation index vs the residual gravity torque linear trend between muscle configurations for each coactivation index. Data points represent mean values for each subject. Lines represent linear fitting of the experimental data. For each muscle configuration, the Pearson’s linear correlation coefficient $\rho$ and its significance level $p$ are shown.

### Main effect of muscle configurations

When comparing the muscle configurations, each coactivation index exhibited a consistent trend of changes across experimental conditions (Fig. 3), as shown by the statistically comparable angular coefficient of the regression lines of the experimental data against the estimated net torque at the shoulder (${TMCf}_{max}$: $F(\text{2,86})=1.03$, $p=0.61$; ${TMCf}_{mean}$: $F(\text{2,86})=1.64$, $p=0.45$; ${TMCf}_{phase}$: $F(\text{2,86})=0.2$, $p=0.99$). The net torque indicates the torque the shoulder flexors have to generate to counteract the action of the force of gravity, thus it was computed as the difference between the arm gravitational torque and the exoskeleton assistance.

### Main effect of exoskeleton assistance

**Fig. 4:**
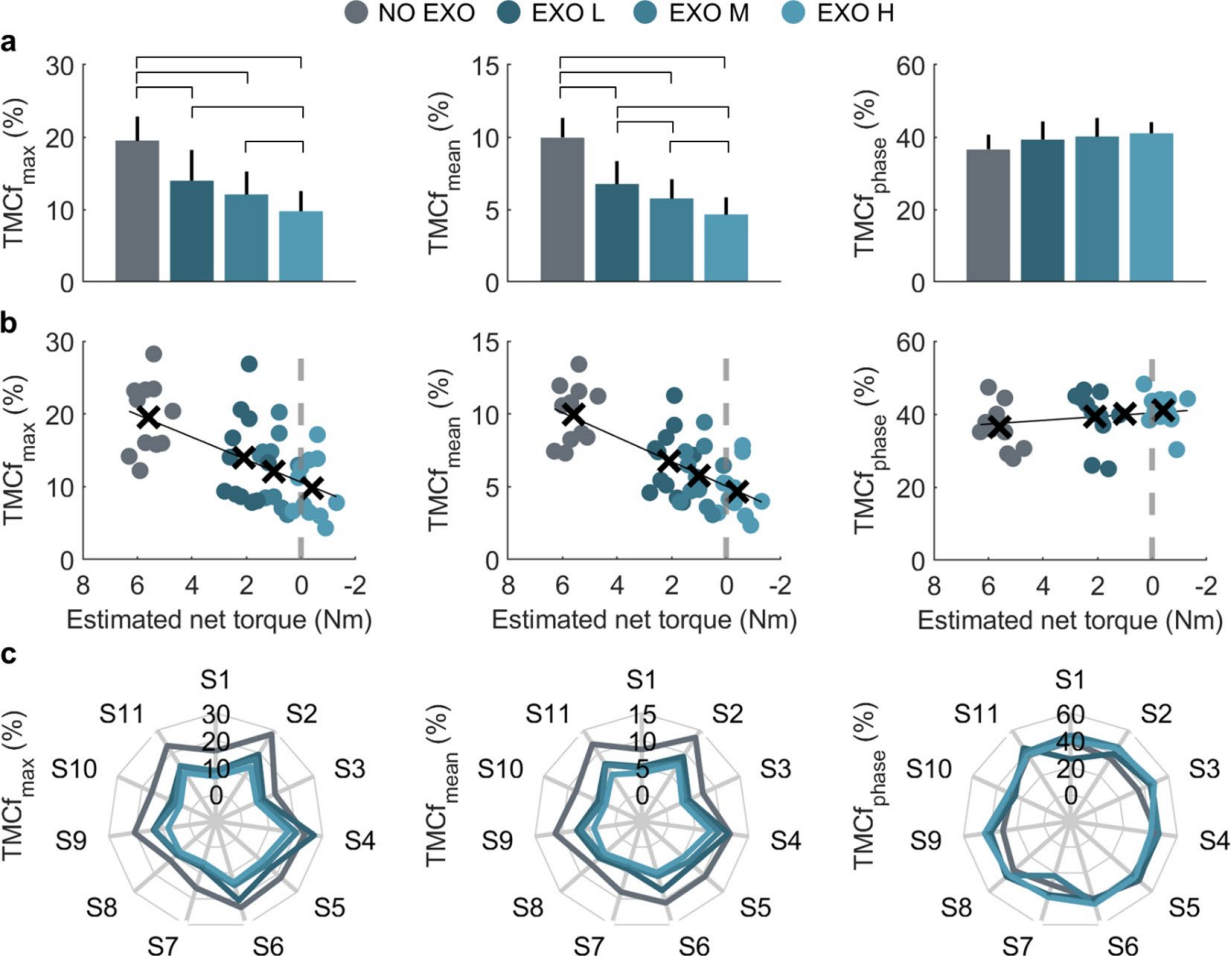
Coactivation indices computed for the tested conditions (NO EXO, EXO L, EXO M, and EXO H) in the 2-muscle configuration (AD vs PD). **(a)** Bar plots represent the mean and 95% CI of the muscle coactivation metrics. Horizontal lines mark statistically significant differences between tested conditions.** (b)** Scatter plots of the estimated net torque vs the coactivation indices. Data points represent mean values for each subject. Mean values for each condition are represented by a black cross. Linear fitting between the net torque and the TMCf indices is shown as a black solid line. **(c)** Spider charts show individual values for the coactivation indices**.**

Since the trend of the changes in each coactivation index is similar between the muscle configurations studied, results are shown only for the 2-muscle configuration, namely for the coactivation due to AD and PD muscles. The peak (${TMCf}_{max}$) and the mean value (${TMCf}_{mean}$) of the coactivation function computed for each sF/E cycle exhibited statistically significant reductions across the experimental conditions (${TMCf}_{max};$ $F(\text{3,30})=56.9$, $p=1.69\cdot {10}^{-12}$; ${TMCf}_{mean}$: $F(\text{3,30})=106.5$, $p=4.3\cdot {10}^{-16}$), as shown in Fig. 4a. The amplitude of the coactivation indices linearly decreased with increasing amount of anti-gravitational support from the exoskeleton, namely with decreasing net torque on the shoulder (Fig. 4b). For both indices, the maximum coactivation reductions compared to the NO EXO were observed in the EXO H condition (Table 1), namely around 51% ($95\% CI \left[-60, -42\right]$, $p = 2.3\cdot {10}^{-6}$) and 54% ($95\% CI [-61, -46]$, $p = 4\cdot {10}^{-6}$) for ${TMCf}_{max}$ and ${TMCf}_{mean}$, respectively.

**Table 1:** Percentage variations of coactivation indices of EXO conditions with respect to the NO EXO, for each muscles configuration.

		${{\varvec{T}}{\varvec{M}}{\varvec{C}}{\varvec{f}}}_{{\varvec{m}}{\varvec{a}}{\varvec{x}}}$			${{\varvec{T}}{\varvec{M}}{\varvec{C}}{\varvec{f}}}_{{\varvec{m}}{\varvec{e}}{\varvec{a}}{\varvec{n}}}$		
		*EXO L*	*EXO M*	*EXO H*	*EXO L*	*EXO M*	*EXO H*
Muscle configuration	2 muscles	− 30 [− 43, − 17]% *p* = 2.6∙10^–3^	− 39 [− 48, − 31]% *p* = 5.2∙10^–6^	− 51 [− 60, − 42]% *p* = 2.3∙10^–6^	− 33 [− 42, − 24]% *p* = 5.2∙10^–5^	− 43 [− 51, − 35]% *p* = 1.4∙10^–6^	− 54 [− 61, − 46]% *p* = 4∙10^–6^
	4 muscles	− 31 [− 41, − 22]% *p* = 1.5∙10^–4^	− 40 [− 48, − 32]% *p* = 3.5∙10^–6^	− 51 [− 61, − 42]% *p* = 1.9∙10^–6^	− 32 [− 41, − 24]% *p* = 1.2∙10^–4^	− 41 [− 50, − 32]% *p* = 1.8∙10^–5^	− 50 [− 62, − 39]% *p* = 1.6∙10^–5^
	6 muscles	− 30 [− 36, − 23]% *p* = 9.1∙10^–6^	− 39 [− 46, − 31]% *p* = 2.1∙10^–6^	− 51 [− 58, − 44]% *p* = 1.9∙10^–7^	− 31 [− 38, − 24]% *p* = 2.2∙10^–5^	− 40 [− 48, − 32]% *p* = 6.8∙10^–6^	− 51 [− 58, − 44]% *p* = 4.2∙10^–7^

Individual results for both the $${TMCf}_{max}$$ and $${TMCf}_{mean}$$ are shown in Fig. 4c, in which the area enclosed by the NO EXO condition line is substantially smaller than the ones relative to the EXO conditions, which are almost comparable. Conversely, the phase of the sF/E cycle corresponding to the coactivation peak ($${TMCf}_{phase})$$ was not significantly affected by the level of assistance provided by the exoskeleton ($$F(\text{3,30})=2,$$ $$p=0.13$$), with an average value falling close to 40% of the sF/E cycle.

## Discussion

Muscle coactivation is a way for the CNS to modulate the mechanical properties of a joint, such as adapting its impedance, in response to changes in task requirements^41^. Despite physiological, excessive muscle coactivation can increase the chance of developing MSDs, especially at the workplace due to the high repetition of dynamic gestures. Although many hypotheses have been made about the neurophysiological mechanisms of muscle coactivation (e.g., the role of spinal and cortical circuitry, the role of cerebellum and basal ganglia), its connection with movement mechanics or through the equilibrium-point hypothesis, a consensus on how the CNS exploits muscle coactivation has not been achieved yet^5^. Nevertheless, muscle coactivation is an ubiquitous phenomenon across different functional, sub-maximal, and maximal activities^1^. While muscle coactivation has been deeply investigated in several domain such as with neurologically-impaired individuals (e.g., people suffering from a stroke)^42^, in studies related to motor control strategies (e.g., in pathological gait and postural stability)^43,44^, and in occupational medicine (e.g., relationship between spine muscle coactivation and development of low-back diseases)^45,46^, currently, the evaluation of muscle coactivation when using an exoskeleton has not been adequately analyzed, yet. Specifically, shoulder muscle coactivation when an upper-limb exoskeleton is used has not been properly studied. In this work, we investigated the effect of a passive upper-limb OE on shoulder muscle coactivation through the analysis of coactivation indices extracted from the $TMCf$ under increasing levels of anti-gravitational support provided by the exoskeleton. Such indices were also compared to those computed in the case the exoskeleton was not worn (NO EXO condition). Furthermore, this study is the first one applying the $TMCf$ to assess the effect of different levels of exoskeleton assistance on muscle coactivation, while previous studies typically focused on exoskeleton-free applications, such as walking^47–49^ or load lifting^35,45,46^.

To investigate the impact of different muscle configurations on muscle coactivation computation, we analyzed various combinations of flexor and extensor muscle groups. When observing the changes of the coactivation indices as a function of the estimated net torque at the shoulder, the consistent trend across all muscle configurations suggests a noteworthy finding. This uniform behavior, regardless of the number of monitored muscles, may imply that, for the sF/E movement, assessing a pair of flexor and extensor muscles (such as the Anterior and Posterior Deltoids) can provide useful information about the impact of the exoskeleton support in shoulder coactivation. The recording of only two muscles may simplify the measurement of muscle activity in more unstructured scenarios, thus potentially fostering the evaluation of muscle coactivation also in field studies, especially when equipping workers with numerous sensors could not be feasible, and reduce workers’ discomfort while testing an exoskeleton during the daily work routine^50^.

Focusing on the 2-muscle configuration, we verified that the use of an exoskeleton, which partly compensates for the arm weight, can reduce muscle coactivation amplitude (i.e., ${TMCf}_{max}$ and ${TMCf}_{mean}$) at the shoulder, and that such a reduction is linearly dependent on the amount of anti-gravitational support delivered. In this regard, we computed the ${TMCf}_{max}$ as a punctual index to gather information on the peak value of concurrent activation of flexor and extensor muscles during the sF/E cycle^45^, which can be associated with peak loads that may cause traumatic damage in the glenohumeral joint, resulting in shoulder disorders, such as rotator cuff tendinopathies or shoulder impingement, and pain^13^. The ${TMCf}_{mean}$, instead, can be associated to the average level of the coactivation across the whole F/E cycle, hence providing information about the gesture execution^45^. From our results, we can hypothesize that the apparent stiffness on the shoulder joint decreased as well, thus potentially unloading the joint from excessive compression and shear forces. However, being the exoskeleton acting in parallel to the human muscles, we can also speculate that, overall, the apparent stiffness of the human-exoskeleton system exhibited no significant variation that would impede movement accuracy, i.e., the effort exerted by muscles to perform the prescribed gesture was reduced, while preserving shoulder tissues and structures^40^.

While amplitude-related indices of coactivation were directly affected by the level of anti-gravitational support the exoskeleton provided, the sF/E phase corresponding to the coactivation peak (${TMCf}_{phase}$) was not influenced. Indeed, the maximum coactivation was observed, in all tested conditions, around the middle part of the sF/E cycle, namely when the maximum load due to gravity is acting on the shoulder joint (namely around 90 deg). This phase also corresponds to the moment in which the shoulder joint transitions the arm motion from an upward to a downward movement (i.e., from flexion to extension), thus decelerating the limb in preparation for the change of the movement direction. Hence, it represents the maximum moment of inertia of the arm for the shoulder muscles to manage. This finding may reflect the fact that the use of the exoskeleton did not considerably alter the movement strategy used by subjects to perform the requested gestures, while simultaneously lowering the muscular effort^40^. Preserving the natural biomechanical movement strategy of an individual wearing an exoskeleton is paramount for seamless human-exoskeleton integration^51^. This may improve efficiency and comfort in the use of the exoskeletons, aligning with the natural biomechanics of the human body, and potentially increase their adoption in real-life contexts^20^.

As required for rigorous biomechanical studies, this research was conducted under highly controlled experimental conditions. Repetitive, well-defined movements were used to reveal the effects of the OE on muscle coactivation indices, minimizing confounding factors from variable conditions. Although the study was not designed to replicate specific work tasks and involved relatively low joint effort, the results indicate a clear influence of the OE on muscle coactivation. These findings invite future research to apply these methods in more realistic settings and movements, where the movement patterns, rhythm, and loading conditions may better reflect specific job activities.

To conclude, the findings of this study have the potential to pave the way for a novel methodology for evaluating upper-limb OEs. This methodology may complement the essential analysis of muscle activation changes across various assistive conditions, including the one in which the exoskeleton is not worn. This approach has the potential to provide valuable insights into the biomechanics of the shoulder joint during occupational tasks, enhancing our understanding beyond the basic assessment of muscle activation. However, incorporating the assessment of shoulder muscle coactivation into existing ergonomics risk assessment methods, such as OCRA or EAWS, is challenging. Future studies could explore the relationship between coactivation indices and ergonomic risk levels quantified by advanced methods, such as the OCRA index. This approach would offer a more comprehensive understanding of the biomechanical risks associated with specific tasks. Furthermore, investigating the coactivation for prolonged periods (e.g., weeks or months) could provide additional insights about middle-to-long term issues in the musculoskeletal system. Looking ahead, while this study focused on a passive exoskeleton, the results obtained may inform the development of human-in-the-loop control strategies for active and semi-active exoskeletons. In such systems, muscle coactivation could be a valuable input for assistive algorithms, guiding the design of strategies aimed at, for example, modulating the muscle coactivation.

Although the results obtained in this study are interesting and potentially relevant for the evaluation of the impact of OEs in repetitive arm gestures, there are some limitations. Indeed, the results refer to the task, the sample, and the exoskeleton used in this study. Hence, changing one of these factors could lead to different findings. Additionally, they are intrinsically limited to the specific monitored muscles and their relative combinations between shoulder flexors and extensors. As also suggested by the authors of the $TMCf$ method used in this study, to obtain consistent results it is fundamental to carefully select the muscles to include in the analysis^4^. In our case, we identified several superficial muscles that are functionally relevant to the executed gesture. Hence, a possible additional limitation of this study is the arbitrary selection of the muscles employed in the computation of the $TMCf$, which could limit the generalizability of the results only to the muscle configurations considered. However, due to the complexity of the glenohumeral joint, which is the most complex joint in the upper body, a comprehensive analysis of muscle coactivation becomes highly difficult, as numerous muscles are involved in its movement. This is one of the reasons the task was intentionally simplified and restricted to shoulder flexion and extension movements in the sagittal plane, ensuring more consistent and comparable kinematics.

The generalizability of the results could also be limited by the small number of subjects and by the tiny population constituted by only female participants. Considering other segments of the population, such as overweight individuals, who may more accurately represent the actual industrial workforce, could offer valuable insights into how muscle coactivation changes when using an exoskeleton. However, it is important to note that estimating muscle activity through surface EMG, and consequently computing coactivation, could be challenged by the presence of fatty tissue, which may affect the accuracy of the measurements^52^. Nevertheless, we believe the results obtained from the current sample are relevant, as the prevalence of work-related MSDs is higher among female workers compared to male workers, with females being 2–5 times more likely to experience repetitive strain injuries^53^.

A further limitation of the study resides in the lack of subjective measures about the participants’ perceived acceptability and comfort of the exoskeleton and their relationship with the changes in the coactivation metrics. Relating such perception-based metrics with muscle coactivation could lead to a deeper evaluation and understanding of the effect of the exoskeleton.

From a future perspective, it would be valuable to assess the presented methodology with actual workers across various application domains (e.g., automotive, manufacturing, logistics) and in more realistic work scenarios. This includes job activities where work gestures are less stereotyped and involve combinations of different sub-tasks, inevitably increasing the possible confounding factors and the related variability. Hence, this study helps to refine the scientific question in preparation for future experiments in more realistic scenarios, such as simulated work tasks or actual workplace environments, where we believe the current methodology can be effectively adapted. Nevertheless, it is important that future studies will consider the design of tasks for which it is possible to identify the associated ergonomic risk by means of, for example, the tools listed in the ISO 11228–3. In this way, we could improve the understanding of the effect an OE can have in real-life environments, potentially accelerating their large-scale adoption^20^.

Finally, investigating muscle coactivation over extended periods (such as during a full work shift), in conjunction also with muscle fatigue^54^, and examining how coactivation patterns change from the beginning to the end of the shift could provide valuable insights into the effects of the exoskeleton on the musculoskeletal system. Such an approach would enhance the generalizability of the outcomes from the present study.

## Methods

### Participants and ethics approval

Eleven healthy female subjects were recruited to take part in the study (age: 25.6 ± 1.4 years, height: 166.7 ± 5 cm, weight: 53.8 ± 3.9 kg, BMI: 19.9 ± 2.3 kg/cm^2^), which was carried out at the premises of the BioRobotics Institute of Scuola Superiore Sant’Anna (Pontedera, Pisa, Italy). Enrolled participants were selected with anthropometric parameters (i.e., height and weight) entailing an estimated anti-gravitational support by the exoskeleton, when set to provide the maximum level of assistance, of about 100% of the arm gravitational torque, according to the methods described in^40^. The experimental procedures were approved by the Institutional Review Board (approval n.24/2022) and were conducted following the principles stated in the Declaration of Helsinki. Before starting the experimental activities, enrolled participants gave their written signed informed consent.

### Passive upper-limb occupational exoskeleton

The exoskeleton used in this study is a commercially-available passive upper-limb OE^55^ (Fig. 2a). It is designed to provide workers with anti-gravitational support to reduce the load on the shoulder joint in physically demanding working activities, such as the ones involving keeping the arms raised overhead for a prolonged time or the execution of repetitive arm gestures. The exoskeleton is a novel version of the one tested by Pacifico and colleagues^56^. It comprises two torque-generator boxes (one per arm), including a spring-based mechanism for assistive torque profile generation, which mimics the arm gravitational torque profile (Fig. 2b). Additionally, the exoskeleton integrates a physical human–machine interface (pHMI) to unload the weight of the device and the reaction forces due to the assistive torque on the user’s pelvis, and a kinematic chain of passive degrees of freedom (pDOFs), to guarantee the self-alignment of the exoskeleton with the human joint axes and ensuring a safe human–machine interaction. By manually changing the pretension of the spring, users can adjust the level of anti-gravitational support over a range of eight discrete values (peak assistive torques range from around 3.5 to 6 Nm). The exoskeleton weighs about 3 kg.

### Study design and experimental procedures

The study consisted of the execution of a repetitive dynamic arm gesture, in which the subjects were requested to perform shoulder flexion and extension movements at a fixed predefined frequency (50 bpm) for 2 min, while holding a light-weighted screwdriver. The movement was performed in the sagittal plane from waist level to overhead and vice-versa, namely covering a range of movement (RoM) of around 100 deg. The fixed RoM and frequency ensured reducing intra- and inter-subject movement variability. These characteristics of the task were necessary to achieve comparable across-subject shoulder kinematics. The task was repeated four times, each time testing a different level of anti-gravitational support, namely NO EXO, EXO L, EXO M, and EXO H. The three levels of exoskeleton assistance were adjusted in order that the exoskeleton compensated about 60%, 80%, and 100% of the arm gravitational torque estimated at the glenohumeral joint^40^. These four trials were pseudo-randomized to avoid order bias (the NO EXO condition was always tested as the first or last). Enough time was given to subjects to rest to avoid effect fatigue, namely one 5-min break between each trial.

Following the SENIAM guidelines^57^, subjects were equipped with EMG sensors (Ag/AgCl bipolar surface electrodes provided by Pirrone & Co., Milan, Italy) to record muscular activities from shoulder superficial muscles employing the BTS FREEEMG 1000 (BTS Bioengineering, Milan, Italy). Monitored muscles were arm flexors, namely the Anterior Deltoid, Medial Deltoid, and Upper Trapezius, and arm extensors, namely the Posterior Deltoid, Latissimus Dorsi, and Triceps Brachii (Fig. 2c). Maximum voluntary contractions (MVCs) were also recorded to normalize data and to allow inter-subject comparison.

Subjects were also equipped with a set of two XSens MTw (XSens, Enschede, The Netherlands) inertial measurement units (IMUs), placed on the right upper arm and sternum, to record the inertial signal of these body segments and to offline estimate the sF/E angle signal. EMG and IMU signals were synchronized through a dedicated device (Trigger Box, BTS Bioengineering, Milan, Italy).

Before starting the testing phase, sufficient time was given to the subjects to familiarize themselves with both the task and the exoskeleton. This phase lasted about 20 min, while the total experiment lasted about 90 min.

Figure 2d depicts the main phases of the experimental protocol. A more detailed description of the experimental procedures can be found in our previous article^40^.

### Data analysis

**Fig. 5:**
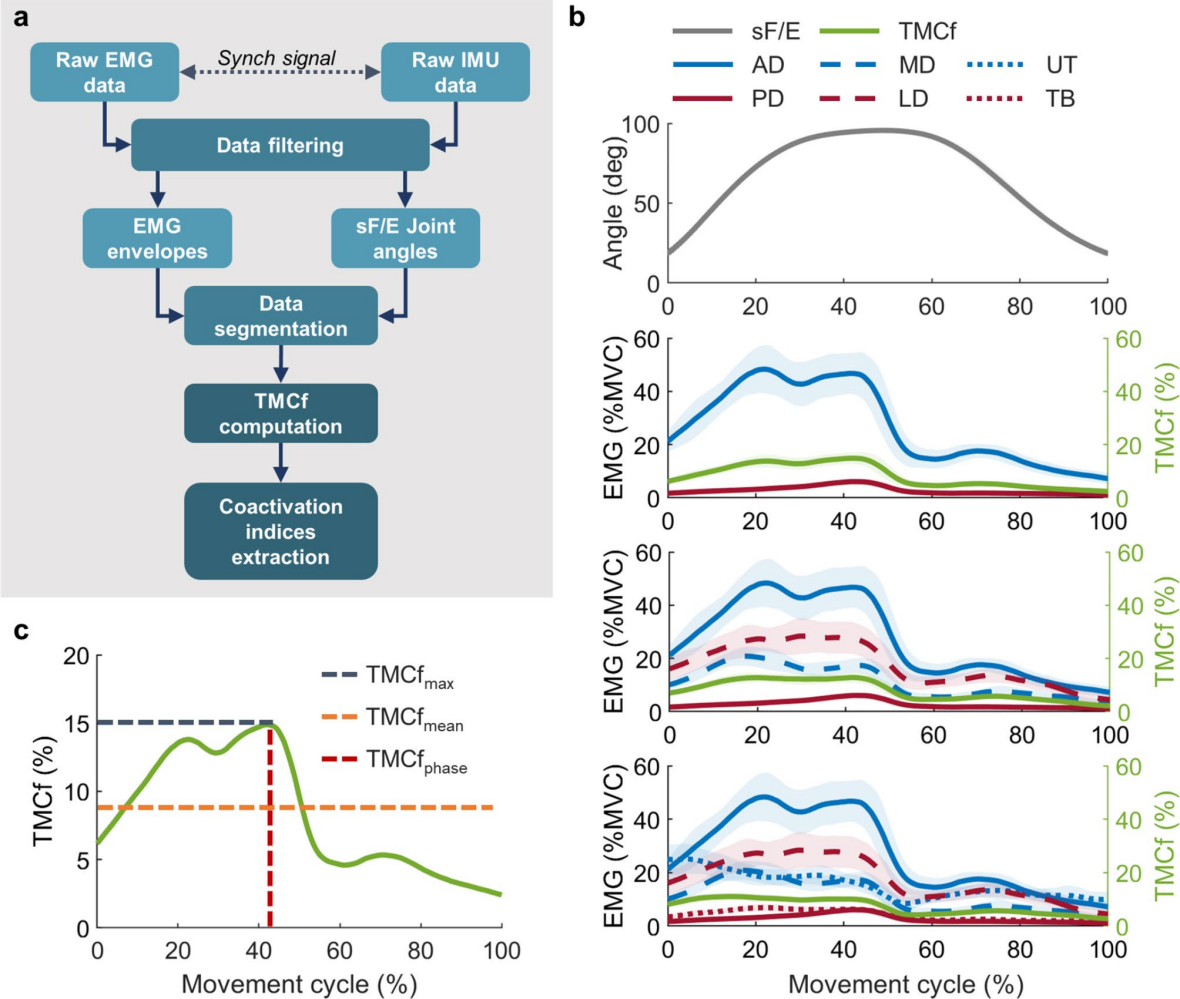
Data processing, coactivation indices computation, and sF/E angle, EMG, and TMCf profiles for a representative subject. **(a)** Data processing flow from raw EMG and IMU data to the extraction of the coactivation indices. **(b)** sF/E angle profiles, EMG, and TMCf profiles are shown for a representative subject. All three muscle configurations are shown. **(c)** Schematic representation of a sample TMCf profile over a movement cycle and identification of the coactivation indices.

Collected data were analyzed offline using custom routines in MATLAB R2019b (The MathWorks, Natick, MA, USA) to compute the $TMCf$ and extract the coactivation indices. The data processing flow is shown in Fig. 5a.

The arm gravitational torque on the shoulder joint was estimated considering the shoulder flexed at 90 deg following the procedure suggested by^58^. The net torque was computed as the difference between the arm gravitational torque and the exoskeleton assistive torque. It is worth noting that, according to the participants’ anthropometry, the estimated net torque corresponded to around 62 ± 5%, 82 ± 7%, and 107 ± 9% of the arm gravitational torque for EXO L, EXO M, and EXO H, respectively.

Raw EMG signals were collected at 1 kHz and processed to obtain the signals linear envelope (4^th^-order band-pass Butterworth filter, cut-off frequencies: 20–400 Hz; rectification; 4^th^-order notch Butterworth filter, cut-off frequency: 50 Hz; zero-lag 100-ms moving average low-pass filter). EMG enveloped signals of each muscle were then amplitude-normalized by their corresponding MVC value. Raw IMU signals were collected at 100 Hz and processed to obtain the Euler angles, representing shoulder angles (i.e., flexion–extension, abduction–adduction, internal–external rotation), from rotation quaternions.

Then, the sF/E angle was used to extract the temporal indices needed to segment the EMG signals into F/E cycles. Every cycle was subsequently time normalized by the cycle duration in the range [0–100]%, representing two consecutive sF/E minima (Fig. 5b).

For each segmented EMG signal, the $TMCf$ was calculated according to (1):

1 $$TMCf\left(d\left(k\right),k\right)=\left(1-\frac{1}{1+{e}^{-12(d\left(k\right)-0.5)}}\right)\cdot \frac{{({\sum }_{m=1}^{M}{EMG}_{m}(k)/M)}^{2}}{{max}_{m=1\dots M}[{EMG}_{m}(k)]}$$

where $d\left(k\right)$ is the mean of the differences between the *k*-th sample of each pair of EMG signals as shown in (2):

2 $$d\left(k\right)=\frac{{\sum }_{m=1}^{M-1}{\sum }_{n=m+1}^{M}|{EMG}_{m}\left(k\right)-{EMG}_{n}\left(k\right)|}{J(M!/(2!\left(M-2\right)!))}$$

where $J$ is the length of the signal (1000 samples in this study) and $M$ is the number of considered muscles (2, 4, 6 in this study). ${EMG}_{m}\left(k\right)$ and ${EMG}_{n}\left(k\right)$ are the *k*-th EMG envelope signal values for the *m*-th and *n*-th muscles, respectively. Figure 5b shows EMG and $TMCf$ profiles over a sF/E cycle for a representative subject for three muscle configurations, namely:

- *2 muscles*: $${\left\{\text{AD}\right\}}_{flexor}$$, $${\left\{\text{PD}\right\}}_{extensor}$$
- *4 muscles*: $${\left\{\text{AD},\text{ MD}\right\}}_{flexor}$$, $${\left\{\text{PD},\text{ LD}\right\}}_{extensor}$$
- *6 muscles*: $${\left\{\text{AD},\text{ MD},\text{ UT}\right\}}_{flexor}$$, $${\left\{\text{PD},\text{ LD},\text{ TB}\right\}}_{extensor}$$

From each $TMCf$ curve, the following muscular coactivation indices have been extracted (Fig. 5c):

- ${TMCf}_{max}$: The peak value of the $TMCf$ curve;
- ${TMCf}_{mean}$: The average value of the $TMCf$ curve;
- ${TMCf}_{phase}$: The percentage of the sF/E cycle corresponding to the ${TMCf}_{max}$.

For each index, the mean value was computed for each subject and then averaged across subjects as mean and 95% CI.

### Statistical analysis

Statistical analysis was performed using custom routines in MATLAB R2019b. For each statistical test performed, a significance level $\alpha =0.05$ was used.

For each coactivation index, in every tested condition and muscle configuration, the data normality assumption was verified using the Shapiro–Wilk’s test. When the data sphericity assumption did not hold using Mauchly’s test, the Epsilon ($\varepsilon$) correction was used (Greenhouse–Geisser correction for $\varepsilon <0.75$ or Huynh–Feldt correction for $\varepsilon \ge 0.75$).

Within each coactivation index, to test the hypothesis of no significant differences in the relationship between the muscle configuration and the estimated net torque, one-way repeated-measures ANOVAs were performed on the angular coefficients of the linear regressions of the experimental data. For each muscle configuration, repeated-measures one-way ANOVAs were used to analyze the effect of the tested conditions on each coactivation index.

When appropriate, *post-hoc* comparisons of the ANOVA levels were tested using the Tukey–Kramer method.

## Data Availability

The data that support the findings of this study are available from the corresponding author upon reasonable request.
